# Methyl 2-[(*tert*-but­oxy­carbon­yl)amino]-3-(3-methyl-2-sulfanyl­idene-2,3-dihydro-1*H*-imidazol-1-yl)propano­ate

**DOI:** 10.1107/S1600536812043486

**Published:** 2012-10-24

**Authors:** Chin-Feng Chan, Yi-Cin Guo, Bor-Hunn Huang, Ming-Jen Chen

**Affiliations:** a Department of Applied Cosmetology and Graduate Institute of Cosmetic Science, Hungkuang University, Taichung 433, Taiwan; b Department of Chemistry, National Chung Hsing University, Taichung 402, Taiwan

## Abstract

In the title compound, C_13_H_21_N_3_O_4_S, the mean plane of the –N(H)—C(=O)—O– group of the carbamate unit forms a dihedral angle of 64.7 (2)° with the mean plane of the –C—C(=O)—O– group of the ester unit. In the crystal, mol­ecules are linked by N—H⋯O=C hydrogen bonds, forming chains along the *c*-axis direction.

## Related literature

The title compound is a mercaptoimidazole derivative. For applications of mercaptoimidazole derivatives in the treatment of hyperpigmentation, see: Kasraee (2002 ▶); Kasraee *et al.* (2005 ▶) and for inhibiting tyrosinase, see: Liao *et al.* (2012 ▶). For typical bond lengths of inter­molecular N—H⋯O=C hydrogen bonds, see: Taylor *et al.* (1984 ▶).
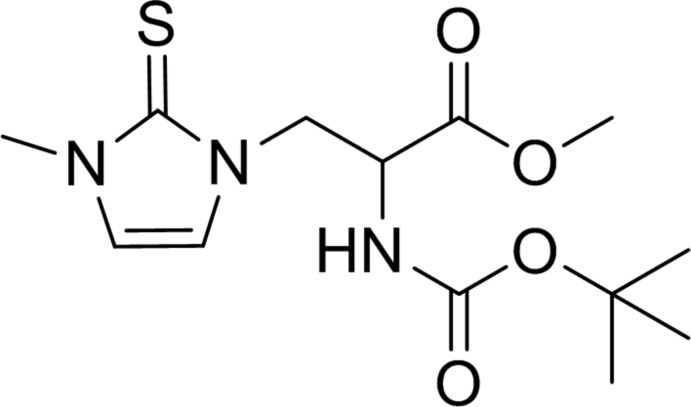


## Experimental

### 

#### Crystal data

C_13_H_21_N_3_O_4_S
*M* *_r_* = 315.39
Monoclinic, 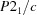

*a* = 8.7636 (1) Å
*b* = 19.1184 (2) Å
*c* = 9.6735 (2) Å
β = 98.485 (1)°
*V* = 1603.02 (4) Å^3^
*Z* = 4
Cu *K*α radiation
μ = 1.97 mm^−1^
*T* = 110 K
0.60 × 0.50 × 0.30 mm

#### Data collection

Agilent Xcalibur (Sapphire3, Gemini) diffractometer
Absorption correction: multi-scan (*CrysAlis PRO*; Agilent, 2010 ▶) *T*_min_ = 0.550, *T*_max_ = 1.000
12262 measured reflections
3093 independent reflections
2923 reflections with *I* > 2σ(*I*)
*R*_int_ = 0.019

#### Refinement

*R*[*F* ^2^ > 2σ(*F* ^2^)] = 0.036
*wR*(*F* ^2^) = 0.137
*S* = 1.05
3093 reflections
190 parameters
H-atom parameters constrained
Δρ_max_ = 0.39 e Å^−3^
Δρ_min_ = −0.53 e Å^−3^

### 

Data collection: *CrysAlis PRO* (Agilent, 2010 ▶); cell refinement: *CrysAlis PRO*; data reduction: *CrysAlis PRO*; program(s) used to solve structure: *SHELXS97* (Sheldrick, 2008 ▶); program(s) used to refine structure: *SHELXL97* (Sheldrick, 2008 ▶); molecular graphics: *SHELXTL* (Sheldrick, 2008 ▶); software used to prepare material for publication: *SHELXL97*.

## Supplementary Material

Crystal structure: contains datablock(s) I, global. DOI: 10.1107/S1600536812043486/lh5544sup1.cif

Structure factors: contains datablock(s) I. DOI: 10.1107/S1600536812043486/lh5544Isup2.hkl

Supplementary material file. DOI: 10.1107/S1600536812043486/lh5544Isup3.cml

Additional supplementary materials:  crystallographic information; 3D view; checkCIF report

## Figures and Tables

**Table 1:** Hydrogen-bond geometry (Å, °)

*D*—H⋯*A*	*D*—H	H⋯*A*	*D*⋯*A*	*D*—H⋯*A*
N3—H3*A*⋯O1^i^	0.88	2.25	2.9819 (14)	140

Symmetry code: (i) 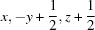.

**Fig. 1.**
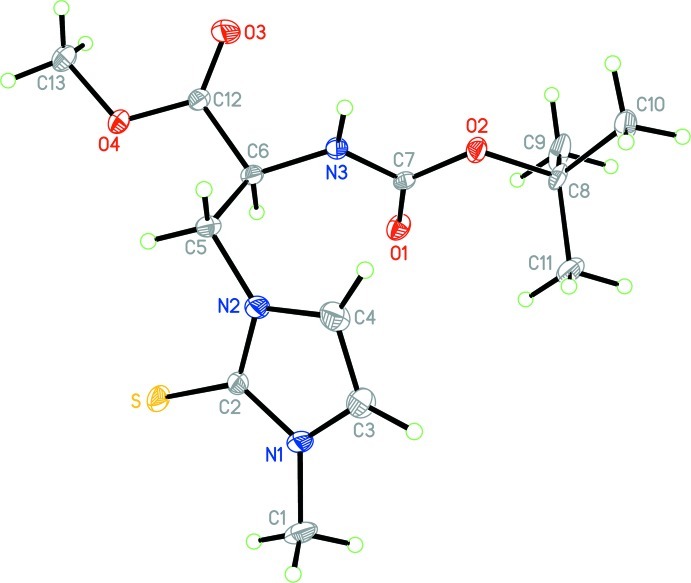
The molecular structure of (I) with ellipsoids for non-H atoms shown at the 50% probability level.

## supplementary crystallographic information

### Comment

Methimazole causes hypopigmentation in some patients during the clinical oral antithyroid medication (Kasraee, 2002; Kasraee *et al.*, 2005). Ergothioneine has a potent inhibition effect on inhibiting tyrosinase enzyme activity, resulting from the presence of the sulfur substituted imidazole ring (Liao *et al.*, 2012). It shows that molecules with a mercaptoimidazole group have potential as depigmenting agents. The title compound (I) is the key intermediate for the synthesis of the amino acid derivatives containing methimazole moiety. Methimazole exists between the 2-thiol and 2-thione forms and has been observed to react in both guizes, depending upon the reaction conditions. Compound (I) is the nitrogen-substituted product from the 2-thione form.

Herein we report the synthesis and crystal structure of the title compound. The molecular structure of (I) is shown in Fig. 1. The essentially planar carbamate group (N3/C7/O1/O2) forms a dihedral angle of 64.7 (2)° with the mean plane of the carboxylate group (C6/C12/O3/O4). In the crystal, molecules are linked by N—H···O═C hydrogen bonds involving the amino and carbamate group forming chains along the *c*-axis direction. Intermolecular N—H···O═C hydrogen bonds are are highlighted in the literature by Taylor *et al.* (1984).

### Experimental

To a mixture of 1-methyl-2-mercaptoimidazole (390 mg, 3.4 mmole) and methyl 3-bromo-2-[(*tert*-butoxycarbonyl)amino]propanoate (970 mg, 3.4 mmol) in 17 ml of *N*,*N*-dimethylformamide was added potassium carbonate (940 mg, 6.8 mmole). The reaction mixture was stirred at 343 K for 1.5 h under N_2_ atmosphere. The resulting mixture was partitioned between dichloromethane (70 ml) and H_2_O (35 ml). The organic layer was dried over MgSO_4_ and concentrated *in vacuo*. The residue was separated by chromatography over silica gel and eluted with hexane/ethyl acetate (3/7) to afford 776 mg of the title compound (I) in 72% yield. Single crystals suitable for X-ray measurements were obtained by recrystallization from a dichloromethane/hexane solution of the title compound at room temperature.

### Refinement

All H atoms were placed in geometrically idealized positions and treated as riding on their parent atoms, with C—H = 0.95 - 1.00 Å, N—H = 0.88 Å and *U*_iso_(H) = 1.2*U*_eq_(C,N).

### Crystal data

**Table:**

C_13_H_21_N_3_O_4_S	-
*M**_r_* = 315.39	*D*_x_ = 1.307 Mg m^−^^3^
Monoclinic, *P*2_1_/*c*	Melting point: 379 K
Hall symbol: -P 2ybc	Cu *K*α radiation, λ = 1.54178 Å
*a* = 8.7636 (1) Å	Cell parameters from 9755 reflections
*b* = 19.1184 (2) Å	θ = 4.6–71.5°
*c* = 9.6735 (2) Å	µ = 1.97 mm^−^^1^
β = 98.485 (1)°	*T* = 110 K
*V* = 1603.02 (4) Å^3^	Cube, colourless
*Z* = 4	0.60 × 0.50 × 0.30 mm
*F*(000) = 672	

### Data collection

**Table:**

Agilent Xcalibur (Sapphire3, Gemini) diffractometer	3093 independent reflections
Radiation source: fine-focus sealed tube	2923 reflections with *I* > 2σ(*I*)
Graphite monochromator	*R*_int_ = 0.019
Detector resolution: 16.0690 pixels mm^-1^	θ_max_ = 71.6°, θ_min_ = 4.6°
ω scans	*h* = −10→9
Absorption correction: multi-scan (*CrysAlis PRO*; Agilent, 2010)	*k* = −23→23
*T*_min_ = 0.550, *T*_max_ = 1.000	*l* = −10→11
12262 measured reflections	

### Refinement

**Table:**

Refinement on *F*^2^	Primary atom site location: structure-invariant direct methods
Least-squares matrix: full	Secondary atom site location: difference Fourier map
*R*[*F*^2^ > 2σ(*F*^2^)] = 0.036	Hydrogen site location: inferred from neighbouring sites
*wR*(*F*^2^) = 0.137	H-atom parameters constrained
*S* = 1.05	*w* = 1/[σ^2^(*F*_o_^2^) + (0.120*P*)^2^] where *P* = (*F*_o_^2^ + 2*F*_c_^2^)/3
3093 reflections	(Δ/σ)_max_ = 0.001
190 parameters	Δρ_max_ = 0.39 e Å^−^^3^
0 restraints	Δρ_min_ = −0.53 e Å^−^^3^

### Special details

**Table:**

Experimental. *CrysAlis PRO*, Oxford Diffraction Ltd., Version 1.171.33.66 (release 28–04-2010 CrysAlis171. NET) (compiled Apr 28 2010,14:27:37) Empirical absorption correction using spherical harmonics, implemented in SCALE3 ABSPACK scaling algorithm.
Geometry. All e.s.d.'s (except the e.s.d. in the dihedral angle between two l.s. planes) are estimated using the full covariance matrix. The cell e.s.d.'s are taken into account individually in the estimation of e.s.d.'s in distances, angles and torsion angles; correlations between e.s.d.'s in cell parameters are only used when they are defined by crystal symmetry. An approximate (isotropic) treatment of cell e.s.d.'s is used for estimating e.s.d.'s involving l.s. planes.
Refinement. Refinement of *F*^2^ against ALL reflections. The weighted *R*-factor *wR* and goodness of fit *S* are based on *F*^2^, conventional *R*-factors *R* are based on *F*, with *F* set to zero for negative *F*^2^. The threshold expression of *F*^2^ > σ(*F*^2^) is used only for calculating *R*-factors(gt) *etc*. and is not relevant to the choice of reflections for refinement. *R*-factors based on *F*^2^ are statistically about twice as large as those based on *F*, and *R*- factors based on ALL data will be even larger.

### Fractional atomic coordinates and isotropic or equivalent isotropic displacement parameters (Å^2^)

**Table:**

	*x*	*y*	*z*	*U*_iso_*/*U*_eq_	
S	0.73233 (4)	0.397097 (16)	0.68939 (3)	0.01934 (17)	
O1	1.13487 (11)	0.21854 (5)	0.76481 (9)	0.0166 (2)	
O2	1.21653 (11)	0.16902 (5)	0.97808 (10)	0.0159 (2)	
O3	1.39031 (11)	0.39024 (5)	0.96950 (12)	0.0242 (3)	
O4	1.20097 (10)	0.46673 (5)	0.89321 (11)	0.0185 (2)	
N1	0.65404 (12)	0.27506 (6)	0.81002 (12)	0.0173 (3)	
N2	0.85617 (12)	0.32536 (6)	0.92269 (12)	0.0154 (3)	
N3	1.18007 (12)	0.28264 (5)	0.96527 (11)	0.0128 (3)	
H3A	1.2174	0.2816	1.0548	0.015*	
C1	0.51211 (16)	0.26296 (8)	0.71401 (16)	0.0257 (3)	
H1A	0.5002	0.2993	0.6417	0.039*	
H1B	0.4240	0.2645	0.7656	0.039*	
H1C	0.5167	0.2170	0.6701	0.039*	
C2	0.74673 (14)	0.33203 (7)	0.80813 (13)	0.0133 (3)	
C3	0.70687 (16)	0.23359 (8)	0.92408 (16)	0.0237 (3)	
H3B	0.6621	0.1909	0.9484	0.028*	
C4	0.83277 (16)	0.26432 (8)	0.99458 (15)	0.0222 (3)	
H4A	0.8938	0.2476	1.0773	0.027*	
C5	0.98306 (14)	0.37429 (7)	0.95850 (14)	0.0150 (3)	
H5A	1.0054	0.3794	1.0613	0.018*	
H5B	0.9531	0.4207	0.9180	0.018*	
C6	1.12846 (13)	0.34846 (6)	0.90267 (13)	0.0124 (3)	
H6A	1.1017	0.3412	0.7996	0.015*	
C7	1.17322 (13)	0.22218 (6)	0.89111 (13)	0.0117 (3)	
C8	1.21603 (16)	0.09602 (6)	0.92637 (15)	0.0155 (3)	
C9	1.32792 (18)	0.08866 (7)	0.82123 (18)	0.0255 (4)	
H9A	1.4312	0.1032	0.8646	0.038*	
H9B	1.2938	0.1183	0.7399	0.038*	
H9C	1.3309	0.0398	0.7914	0.038*	
C10	1.27314 (18)	0.05494 (7)	1.05848 (16)	0.0228 (3)	
H10A	1.1998	0.0602	1.1251	0.034*	
H10B	1.3743	0.0728	1.1004	0.034*	
H10C	1.2821	0.0054	1.0351	0.034*	
C11	1.05181 (16)	0.07481 (7)	0.86891 (15)	0.0215 (3)	
H11A	0.9859	0.0809	0.9415	0.032*	
H11B	1.0502	0.0257	0.8399	0.032*	
H11C	1.0135	0.1042	0.7882	0.032*	
C12	1.25754 (15)	0.40293 (6)	0.92748 (14)	0.0131 (3)	
C13	1.31275 (16)	0.52315 (7)	0.90481 (17)	0.0236 (3)	
H13A	1.2600	0.5673	0.8774	0.035*	
H13B	1.3897	0.5137	0.8432	0.035*	
H13C	1.3641	0.5266	1.0017	0.035*	

### Atomic displacement parameters (Å^2^)

**Table:**

	*U*^11^	*U*^22^	*U*^33^	*U*^12^	*U*^13^	*U*^23^
S	0.0231 (3)	0.0120 (2)	0.0207 (3)	−0.00343 (11)	−0.00411 (17)	0.00477 (11)
O1	0.0231 (5)	0.0148 (5)	0.0116 (5)	−0.0038 (3)	0.0021 (4)	−0.0014 (3)
O2	0.0216 (5)	0.0103 (5)	0.0145 (5)	−0.0009 (3)	−0.0014 (4)	−0.0014 (3)
O3	0.0128 (5)	0.0217 (5)	0.0364 (6)	−0.0026 (4)	−0.0020 (4)	0.0038 (4)
O4	0.0153 (5)	0.0110 (5)	0.0281 (6)	−0.0027 (3)	−0.0001 (4)	−0.0003 (4)
N1	0.0109 (5)	0.0163 (6)	0.0243 (6)	−0.0033 (4)	0.0010 (4)	0.0038 (4)
N2	0.0115 (5)	0.0145 (6)	0.0198 (6)	−0.0016 (4)	0.0011 (4)	0.0042 (4)
N3	0.0147 (5)	0.0121 (5)	0.0108 (5)	0.0002 (4)	−0.0008 (4)	−0.0005 (4)
C1	0.0149 (7)	0.0260 (8)	0.0345 (9)	−0.0096 (5)	−0.0017 (6)	0.0024 (6)
C2	0.0113 (6)	0.0110 (6)	0.0178 (7)	0.0000 (4)	0.0031 (5)	−0.0004 (4)
C3	0.0182 (7)	0.0200 (7)	0.0335 (8)	−0.0042 (5)	0.0055 (6)	0.0119 (6)
C4	0.0175 (7)	0.0226 (7)	0.0264 (8)	−0.0010 (5)	0.0031 (5)	0.0125 (6)
C5	0.0114 (6)	0.0156 (7)	0.0173 (7)	−0.0015 (5)	0.0002 (5)	−0.0019 (5)
C6	0.0116 (6)	0.0129 (6)	0.0122 (6)	−0.0015 (4)	−0.0002 (5)	−0.0007 (5)
C7	0.0078 (5)	0.0122 (6)	0.0155 (6)	−0.0027 (4)	0.0022 (4)	0.0004 (5)
C8	0.0208 (7)	0.0078 (6)	0.0179 (7)	−0.0042 (5)	0.0028 (6)	−0.0017 (5)
C9	0.0321 (8)	0.0118 (6)	0.0366 (9)	−0.0032 (6)	0.0184 (7)	−0.0024 (6)
C10	0.0297 (7)	0.0129 (6)	0.0242 (7)	−0.0019 (5)	−0.0014 (6)	0.0028 (5)
C11	0.0231 (7)	0.0198 (7)	0.0200 (7)	−0.0105 (5)	−0.0019 (5)	0.0023 (5)
C12	0.0139 (6)	0.0127 (6)	0.0126 (6)	−0.0018 (4)	0.0010 (5)	−0.0008 (4)
C13	0.0189 (7)	0.0137 (6)	0.0369 (9)	−0.0064 (5)	−0.0004 (6)	0.0010 (6)

### Geometric parameters (Å, º)

**Table:**

S—C2	1.6852 (13)	C4—H4A	0.9500
O1—C7	1.2204 (16)	C5—C6	1.5370 (16)
O2—C7	1.3380 (16)	C5—H5A	0.9900
O2—C8	1.4825 (14)	C5—H5B	0.9900
O3—C12	1.1993 (17)	C6—C12	1.5301 (16)
O4—C12	1.3398 (15)	C6—H6A	1.0000
O4—C13	1.4502 (16)	C8—C11	1.5193 (18)
N1—C2	1.3605 (16)	C8—C9	1.5199 (19)
N1—C3	1.3819 (18)	C8—C10	1.5204 (19)
N1—C1	1.4567 (17)	C9—H9A	0.9800
N2—C2	1.3604 (17)	C9—H9B	0.9800
N2—C4	1.3890 (17)	C9—H9C	0.9800
N2—C5	1.4553 (16)	C10—H10A	0.9800
N3—C7	1.3571 (16)	C10—H10B	0.9800
N3—C6	1.4402 (16)	C10—H10C	0.9800
N3—H3A	0.8800	C11—H11A	0.9800
C1—H1A	0.9800	C11—H11B	0.9800
C1—H1B	0.9800	C11—H11C	0.9800
C1—H1C	0.9800	C13—H13A	0.9800
C3—C4	1.344 (2)	C13—H13B	0.9800
C3—H3B	0.9500	C13—H13C	0.9800
			
C7—O2—C8	121.10 (10)	C5—C6—H6A	108.1
C12—O4—C13	115.91 (10)	O1—C7—O2	126.64 (11)
C2—N1—C3	109.86 (11)	O1—C7—N3	124.20 (11)
C2—N1—C1	125.02 (12)	O2—C7—N3	109.16 (11)
C3—N1—C1	124.88 (12)	O2—C8—C11	109.28 (11)
C2—N2—C4	110.38 (11)	O2—C8—C9	110.05 (10)
C2—N2—C5	123.76 (11)	C11—C8—C9	113.60 (12)
C4—N2—C5	125.80 (11)	O2—C8—C10	102.62 (11)
C7—N3—C6	122.36 (10)	C11—C8—C10	110.26 (11)
C7—N3—H3A	118.8	C9—C8—C10	110.47 (12)
C6—N3—H3A	118.8	C8—C9—H9A	109.5
N1—C1—H1A	109.5	C8—C9—H9B	109.5
N1—C1—H1B	109.5	H9A—C9—H9B	109.5
H1A—C1—H1B	109.5	C8—C9—H9C	109.5
N1—C1—H1C	109.5	H9A—C9—H9C	109.5
H1A—C1—H1C	109.5	H9B—C9—H9C	109.5
H1B—C1—H1C	109.5	C8—C10—H10A	109.5
N2—C2—N1	105.34 (11)	C8—C10—H10B	109.5
N2—C2—S	126.68 (10)	H10A—C10—H10B	109.5
N1—C2—S	127.98 (10)	C8—C10—H10C	109.5
C4—C3—N1	107.92 (13)	H10A—C10—H10C	109.5
C4—C3—H3B	126.0	H10B—C10—H10C	109.5
N1—C3—H3B	126.0	C8—C11—H11A	109.5
C3—C4—N2	106.51 (12)	C8—C11—H11B	109.5
C3—C4—H4A	126.7	H11A—C11—H11B	109.5
N2—C4—H4A	126.7	C8—C11—H11C	109.5
N2—C5—C6	110.72 (10)	H11A—C11—H11C	109.5
N2—C5—H5A	109.5	H11B—C11—H11C	109.5
C6—C5—H5A	109.5	O3—C12—O4	124.96 (12)
N2—C5—H5B	109.5	O3—C12—C6	124.98 (11)
C6—C5—H5B	109.5	O4—C12—C6	110.05 (10)
H5A—C5—H5B	108.1	O4—C13—H13A	109.5
N3—C6—C12	110.47 (10)	O4—C13—H13B	109.5
N3—C6—C5	110.99 (10)	H13A—C13—H13B	109.5
C12—C6—C5	110.99 (10)	O4—C13—H13C	109.5
N3—C6—H6A	108.1	H13A—C13—H13C	109.5
C12—C6—H6A	108.1	H13B—C13—H13C	109.5

### Hydrogen-bond geometry (Å, º)

**Table:**

*D*—H···*A*	*D*—H	H···*A*	*D*···*A*	*D*—H···*A*
N3—H3*A*···O1^i^	0.88	2.25	2.9819 (14)	140

Symmetry code: (i) *x*, −*y*+1/2, *z*+1/2.
